# Genome Sequences of Four Potentially Therapeutic Bacteriophages Infecting Shiga Toxin-Producing Escherichia coli

**DOI:** 10.1128/MRA.00749-20

**Published:** 2020-09-03

**Authors:** Carla Dias, Carina Almeida, Małgorzata Łobocka, Hugo Oliveira

**Affiliations:** a CEB–Centre of Biological Engineering, University of Minho, Braga, Portugal; b INIAV, IP–National Institute for Agrarian and Veterinary Research, Vairão, Vila do Conde, Portugal; c Department of Microbial Biochemistry, Institute of Biochemistry and Biophysics of the Polish Academy of Sciences, Warsaw, Poland; Queens College

## Abstract

Four phages infecting Shiga toxin-producing Escherichia coli (STEC) strains of different serotypes were isolated from wastewater samples. Their virion DNAs range from 51 to 170 kbp, are circularly permuted or have defined terminal repeats, and can encode 82 to 279 proteins. Despite their high similarity to other phages, only about 30% of their genes have a predicted function.

## ANNOUNCEMENT

Shiga toxin-producing Escherichia coli (STEC) causes significant foodborne diseases in humans. Being generally nonpathogenic in ruminants, they use their gut as a natural reservoir. Transmission to humans occurs through the consumption of contaminated foods, such as raw or undercooked meat products, raw milk, and contaminated raw vegetables. Because fecal shedding is the major contamination source of carcasses, causing subsequent food recalls and human outbreaks, the role of the live animal in the production of a safe food product is critical. Here, we report the isolation of four broad STEC-infecting phages (vB_EcoM_Lutter [Lutter], vB_EcoM_Ozark [Ozark], vB_EcoM_Gotham [Gotham], and vB_EcoS_Chapo [Chapo]) isolated in Braga, Portugal.

Phages were isolated and produced as described previously (1). Briefly, sewage samples enriched with double-strength tryptic soy broth medium and STEC strains were grown overnight at 37°C with agitation. Filtered supernatants were spotted onto bacterial lawns, and collected phages were used for further purification.

Phage genomic DNA was extracted using phenol-chloroform-isoamyl alcohol extraction (2). Next, whole-genome libraries were constructed using a TruSeq Nano DNA library prep kit. The generated DNA fragments were multiplexed and sequenced in the same Illumina MiSeq run using 300-bp paired-end sequencing reads. The sequence reads were assembled in the Geneious Prime 2020 (Biomatters Ltd., New Zealand) *de novo* assembler (with medium-low sensitivity), yielding average coverages of 97× (61,819 reads), 20× (9,253 reads), 79× (31,782 reads), and 130× (19,306 reads) for Lutter, Ozark, Gotham, and Chapo, respectively. Quality control of the sequence reads was performed with FastQC v0.11.5 (3), while the assembly quality was verified with Geneious Prime (4). The assembled reads of Lutter, Ozark, and Chapo formed single contigs of overlapping ends with no regions of 2× increased coverage, as expected in the case of terminally redundant and circularly permuted sequences. Their starts were selected to align with the starts of the genomes of similar reference phages. The genomes were annotated using MyRAST (5), BLAST (6), tRNAscan-SE v2.0 (7), ARAGORN (8), PhagePromoter (9), and HHpred (10) (with default program parameters) and manually inspected. A summary of their basic characteristics is presented in Table 1.

**TABLE 1:** Morphology and overall features of isolated *Escherichia* phages

Phage name	Morphology (family)	Subfamily, genus	Genome size (bp)	Virion DNA	Packaging strategy	G+C content (%)	No. of CDS^*a*^	No. of tRNAs
vB_EcoM_Lutter	*Myoviridae* (*Myoviridae*)	*Tevenvirinae*, *Tequatrovirus*	170,054	Terminally redundant, circularly permuted	Headful packaging, preferred *pac* cuts between pos.^*b*^ 97225 and 97248 of genomic sequence	35.4	279	8
vB_EcoM_Ozark	*Myoviridae* (*Myoviridae)*	*Tevenvirinae*, *Tequatrovirus*	167,600	Terminally redundant, circularly permuted	Headful packaging, preferred *pac* cuts between pos.^*b*^ 94420 and 94443 of genomic sequence	39.5	268	10
vB_EcoM_Gotham	*Myoviridae* (*Myoviridae*)	*Vequintavirinae*, *Vequintavirus*	137,054	With 459-bp terminal repeats	Same specific start sequence for packaging of all virions	43.7	214	6
vB_EcoS_Chapo	*Siphoviridae* (*Drexlerviridae*)	*Tunavirinae*, *Tunavirus*	51,099	Terminally redundant, circularly permuted	Headful packaging, *pac* cut at pos.^*b*^ 68/69 of genomic sequence	45.5	82	0

a CDS, coding DNA sequences.

b pos., position(s).

Lutter was isolated using a STEC O104 strain. It is a myovirus with a 170,054-bp genome that can encode 279 putative proteins (only 120 with predicted function) and shares 90% overall nucleotide identity with the *Escherichia* phage teqhad (GenBank accession number MN895434). Ozark, isolated using a different STEC O29:H12 strain, is closely related to Lutter (97% overall nucleotide identity). They are both related to prototypical phage T4 and share the preferred 24-bp region of T4 DNA packaging. Gotham is a smaller myovirus with a 137,025-bp DNA molecule and 459-bp terminal repeats, sharing 90% overall nucleotide identity with several other *Escherichia* phages (e.g., vB_EcoM-ECP26, GenBank accession number MK883717). Chapo is a siphovirus isolated using the STEC O29:H12 strain and is related to phage T1. It has a 51,099-bp genome divided into oppositely transcribed halves and can encode 82 potential proteins (only 22 with predicted functions). The *pac* cut site of Chapo was localized between positions 68 and 69 of the genomic sequence pointed out by the identical ends in ∼20% of these region reads. All the genomes have defined modules coding different functions. In particular, the lysis cassettes of the myoviruses contain putative holin and endolysin genes that are separated, with the exception of Gotham, where the holin gene was not identified. Siphovirus Chapo is predicted to encode a holin, an endolysin, and u-spanin canonical genes.

### Data availability.

The GenBank accession numbers are MT682713, MT682714, MT682715, and MT682716 for vB_EcoM_Ozark, vB_EcoM_Lutter, vB_EcoS_Chapo, and vB_EcoM_Gotham, respectively. The corresponding SRA data have been deposited in NCBI under BioProject accession number PRJNA646048.
